# Soziale Arbeit im Ausnahmezustand?! Professionstheoretische Forschungsnotizen zur Corona-Pandemie

**DOI:** 10.1007/s12592-020-00347-0

**Published:** 2020-06-19

**Authors:** Christina Buschle, Nikolaus Meyer

**Affiliations:** 1 Arbeitsbereich Erwachsenenpädagogik, IUBH Internationale Hochschule Fernstudium, Kaiserplatz 1, 83435 Bad Reichenhall, Deutschland; 2 Fachbereich Sozialwesen, Hochschule Fulda, Leipziger Straße 123, 36037 Fulda, Deutschland

**Keywords:** Corona-Pandemie, Soziale Folgen, Gesellschaftliche Anerkennung, Professionalisierung, Soziale Arbeit, Corona pandemic, Social impact, Social recognition, Professionalization, Social work

## Abstract

Die Corona-Pandemie verändert aktuell zahlreiche Lebens- und Arbeitsbereiche in Deutschland. Im vorliegenden Beitrag werden erste Trends aus einer bundesweiten Online-Befragung mit 1867 Beschäftigten der Sozialen Arbeit zu den Auswirkungen der Einschränkungen auf die Beschäftigungssituation in der Sozialen Arbeit vorgestellt. Deutlich wird bereits jetzt: Neben Arbeitsverdichtungen nehmen die Beschäftigten veränderte Arbeitsbündnisse mit den Adressat*innen ebenso wie sich wandelnde professionelle Standards wahr – bei gleichzeitig mangelnder gesellschaftlicher Anerkennung. Die Folgen der Corona-Pandemie für die Soziale Arbeit sind zum aktuellen Zeitpunkt noch nicht absehbar, münden für die Mehrheit der Befragten aber in größeren Anforderungen an das eigene Handlungsfeld.

## Abstract (en)

The corona pandemic is currently transforming many areas of life and work in Germany. This article presents first results from a nationwide online survey. In this survey, 1867 employees of social work were asked about the consequences of restrictions on the employment situation in the area of social work. It is already clear: In addition to the intensification of work, employees perceive changes in the working bonds with their clients as well as changing professional standards, while, at the same time, witnessing a lack of social recognition. The consequences of the corona pandemic for social work are currently not foreseeable; yet the majority of the respondents expect the requirements within their own field of work to increase.

## Einleitung

Die Ausbreitung des Coronavirus SARS-CoV‑2 und die damit verbundene Erkrankung COVID-19 verändert aktuell zahlreiche Lebens- und Arbeitsbereiche in Deutschland. Hier und weltweit stehen damit besonders auch die Beschäftigten in der Sozialen Arbeit im Fokus, denn dieser Bereich nimmt in der Krisenbewältigung eine zentrale Funktion ein. Darauf weist die, aus professionstheoretischer Perspektive bedeutsame^1^, internationale Berufsgruppenvertretung International Federation of Social Workers (IFSW) hin: „Zu den Schlüsselfunktionen der Sozialarbeit […] gehören:

1. Sicherstellen, dass die am stärksten gefährdeten Personen in die Planung und Reaktion einbezogen werden.
2. Organisation von Gemeinschaften, um sicherzustellen, dass das Nötigste wie Nahrung und sauberes Wasser verfügbar ist.
3. In sozialen Diensten und in politischen Umgebungen dafür eintreten, dass sich die Dienste anpassen, offen und proaktiv bei der Unterstützung von Gemeinschaften und schutzbedürftigen Bevölkerungsgruppen bleiben.
4. Erleichterung der physischen Distanzierung und der sozialen Solidarität.
5. Als Beruf für die Förderung und Stärkung der Gesundheits- und Sozialdienste als wesentlichen Schutz gegen das Virus, die Ungleichheit und die daraus resultierenden sozialen und wirtschaftlichen Herausforderungen eintreten“ (IFSW 2020).

Ähnliche Stellungnahmen – verbunden mit Hinweisen auf die prekären Lebenssituationen der Adressat*innen sowie der sich durch die Pandemie verändernden Arbeitsbedingungen der Beschäftigten – sind in den vergangen Wochen von zahlreichen Fach- und Berufsverbänden in unterschiedlichen Nuancierungen mit ungleicher medialer Wirkung publiziert worden (vgl. Arbeitsgemeinschaft für Kinder- und Jugendhilfe (AGJ) 2020; Bundesarbeitsgemeinschaft Wohnungslosenhilfe (BAG W) 2020; Deutscher Berufsverband Soziale Arbeit (DBSH) 2020; Deutsche Vereinigung für Soziale Arbeit im Gesundheitswesen (DVSG) 2020; Kinderschutzbund 2020)^2^. Dabei wird deutlich: Die Corona-Pandemie und die mit ihr einhergehenden Hygienemaßnahmen wirken sich auf die Tätigen in der Sozialen Arbeit und deren professionelle Arbeitsbeziehungen aus. So beschreiben die Stellungnahmen der Fach- und Berufsverbände immer wieder die Gefahr veränderter Handlungen mit Blick auf verschobene Aufträge, nicht mehr anwendbare und dabei eigentlich geeignete Technologien (vgl. Nittel et al. 2020), fehlende Absprachen mit Vorgesetzten, den Wegfall von Fallbesprechungen oder anderer Angebote des Austauschs im sozialpädagogischen Team. Auf diese Weise gerät das professionelle Arbeitsbündnis zumindest kurzfristig in Bedrängnis (vgl. Oevermann 1996)^3^.

Derzeit ist allerdings völlig offen, welche langfristigen Veränderungen in der professionellen Handlung und Haltung die Corona-Pandemie sowie die damit verbundenen Verordnungen und Vorschriften in der Kontaktaufnahme tatsächlich stattfinden. Unmittelbare Veränderungen zeigen sich aber bereits jetzt. Um einen Einblick in das subjektive Erleben der eigenen Beschäftigungssituation in der Sozialen Arbeit in Folge der Corona-Pandemie zu erhalten, entstand die nachfolgend vorgestellte Befragung „Corona und die Folgen für die Soziale Arbeit“. Zunächst wird das empirische Vorgehen kurz erläutert (2) und erste Trends aus der quantitativen und qualitativen Analyse vorgestellt (3). Das Fazit schließt mit vorläufigen Überlegungen zu einer professionstheoretischen Verortung (4). Immerhin, das wird die Analyse noch zeigen, scheinen die Folgen der Corona-Pandemie die Soziale Arbeit erst noch zu treffen.

## Empirisches Vorgehen

Ziel der Befragung war es, eine Momentaufnahme zu den Auswirkungen von Einschränkungen auf die Beschäftigungssituation in der Sozialen Arbeit nach ausgewählten Kriterien abzubilden, und zwar zum Zeitpunkt der bis dato härtesten Maßnahmen in Folge der Corona-Pandemie. Die Befragung wurde als Online-Erhebung^4^ konzipiert und vom 7. bis 15. April 2020 als Link über ausgewählte Multiplikator*innen^5^ unter den Beschäftigten des Arbeitsfeldes gestreut. Die Ergebnisse sind dabei nicht repräsentativ, d. h. es können keine verallgemeinernden Rückschlüsse auf die Grundgesamtheit der Beschäftigten in der Sozialen Arbeit gezogen, sondern nur Aussagen über Personen, die den Fragebogen ausgefüllt haben, getätigt werden. Insgesamt liegen 1867 verwertbare Fragebögen vor^6^.

Aufgrund der zeitlichen Einschränkungen (Lockerungen der Kontaktbeschränkungen ab dem 16.04.2020 standen im Raum) und nicht vorhandener Fördermittel mussten im Rahmen der Vorbereitung der Studie und der Konstruktion des Fragenbogens Entscheidungen getroffen werden, die zu Lasten des empirischen Gehalts gehen. Außerdem waren methodische Kompromisse sowie eine themenbezogene Reduktion notwendig. Vor diesen Hintergrund dienten die offenen Antwortmöglichkeiten (bei vier Fragen) der inhaltlichen Differenzierung und gaben den Teilnehmenden die Gelegenheit, ergänzende Schwerpunkte zu setzen. Die offenen Antworten wurden angelehnt an die qualitative Inhaltsanalyse nach Udo Kuckartz (2016) ausgewertet und in Beziehung zu den weiteren Erkenntnissen gesetzt.

Nachfolgend werden erste Trends der Befragung beschrieben. Eine ausdifferenzierte Betrachtung der Folgen der Corona-Pandemie für die Soziale Arbeit wird zu einem späteren Zeitpunkt erfolgen.

## Soziale Arbeit in Zeiten von Corona: erste Trends

Die 1867 Teilnehmenden an der Befragung kommen aus dem gesamten Bundesgebiet, vor allem aber aus den Bundesländern Bayern (22 %), Nordrhein-Westfalen (16 %), Hessen (11 %) und Niedersachsen (11 %). Als Geschlechtszuordnung haben 78 % der Teilnehmenden weiblich angegeben, 21 % männlich und 1 % divers. Der Großteil der Befragten ist in einem Alter zwischen 25 und 34 Jahren (39 %). Ein knappes Drittel der Teilnehmenden war zum Zeitpunkt der Befragung im öffentlichen Dienst angestellt, bei einem kirchlichen Träger (23 %), einer privatwirtschaftlichen Institution (21 %) oder bei einem Träger der Wohlfahrtspflege (20 %). 2 % der Teilnehmenden sind selbstständig bzw. freiberuflich tätig, verbeamtet (2 %) oder weiteres (2 %). Bei 9 % der berücksichtigten Teilnehmenden (*n* = 1838) hat sich der Beschäftigungsstatus im Rahmen der ersten Phase der Pandemie verändert.

**Figure 1:**
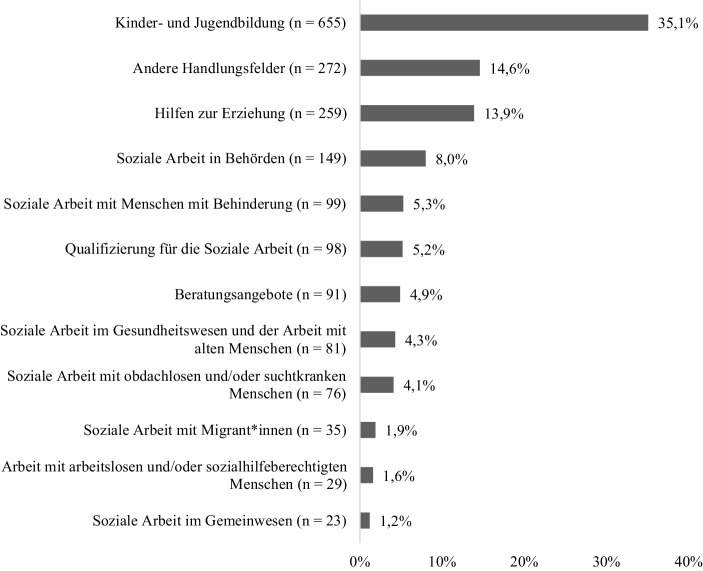

Insgesamt sind eine Vielzahl an Handlungsfeldern der Sozialen Arbeit in der Erhebung vertreten (siehe Abb. 1, Differenzierung in Anlehnung an Thole 2012; Meyer und Siewert 2020), davon mit 35,1 % Beschäftigte im Bereich der Kinder- und Jugendbildung (v. a. in Kindertageseinrichtungen) am stärksten, gefolgt vom Handlungsfeld Hilfen zur Erziehung (13,9 %, v. a. Heimerziehung) und Sozialer Arbeit in Behörden (8 %, v. a. Allgemeiner Sozialer Dienst).^7^

Knapp 60 % der Befragten (*n* = 1862) haben angegeben, dass die eigene Einrichtung bzw. der Dienst geöffnet bzw. aufrecht erhalten bleibt, und zwar sowohl für Mitarbeiter*innen als auch für Adressat*innen. Von diesen (*n* = 1082) sind es mit 31 % vor allem die Einrichtungen der Kinder- und Jugendbildung und die Hilfen zur Erziehung (22 %), die sowohl für Mitarbeiter*innen als auch für Adressat*innen geöffnet haben. Ebenso Behörden (7 %) oder Einrichtungen für die Arbeit mit Menschen mit Behinderungen (7 %). Teilweise geöffnet haben 31 % der Einrichtungen und gänzlich geschlossen sind nach Angaben der Befragten (*n* = 1862) insgesamt 9 % der Einrichtungen, wobei letzteres im Vergleich zwischen den Handlungsfeldern (*n* = 170) vor allem für Einrichtungen der Kinder- und Jugendbildung (56 %), aber auch mit 21 % für die Arbeit im Rahmen der Qualifizierung für die Soziale Arbeit zutrifft. Über alle Handlungsfelder hinweg sind 7 % der Befragten (*n* = 1817) in der Folge von einer Form der Stundenregulierung oder der Anmeldung von Kurzarbeit betroffen.

**Figure 2:**
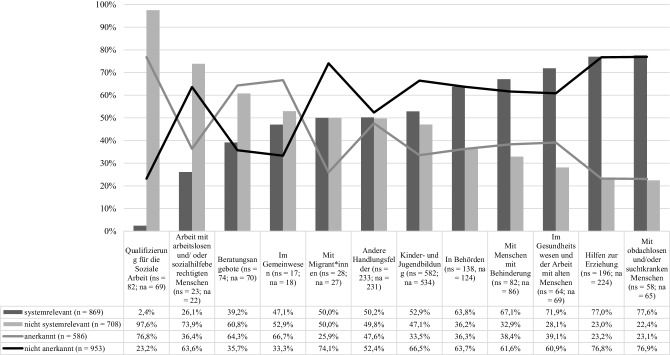

Darüber hinaus konnten die Befragten angeben, ob ihre Tätigkeit offiziell als systemrelevant eingestuft^8^ wurde. Dies bestätigten 55 % der Befragten (*n* = 1577), sie kommen vor allem aus den Handlungsfeldern der Sozialen Arbeit in Behörden, der Hilfen zur Erziehung, dem Gesundheitswesen und der Arbeit mit alten Menschen, mit Menschen mit Behinderung sowie mit obdachlosen und/oder suchtkranken Menschen. Die Uneinheitlichkeit der Einstufungen als „systemrelevant“ in den von der Umfrage abgedeckten Bereichen ist allerdings erheblich^9^. Sie reicht von nur etwas mehr als 2 % im Bereich Ausbildung (Qualifizierung für Soziale Arbeit) bis zu fast 80 % im Bereich der Arbeit mit Obdachlosen und/oder suchtkranken Menschen. Diese Unterschiede werden im Balkendiagramm in den beiden Ausprägungen „systemrelevant“ und „nicht systemrelevant“ in Abb. 2 deutlich.

Neben der Frage nach dem offiziellen Status der Systemrelevanz, wurde auch die Wahrnehmung der beruflichen Anerkennung der eigenen Tätigkeit erhoben^10^. In Abb. 2 wird diese durch das Liniendiagramm mit den beiden Ausprägungen „anerkannt“ und „nicht anerkannt“ abgebildet^11^. Unterschiede zwischen den Handlungsfeldern ergeben sich auch hier. Während die wahrgenommene gesellschaftliche Anerkennung für Beschäftigte im Bereich der Ausbildung (Qualifizierung für die Soziale Arbeit) mit 77 % am höchsten ist, liegt sie mit jeweils 23 % im Bereich der Arbeit mit Obdachlosen und/oder suchtkranken Menschen und in den Hilfen zur Erziehung sowie mit 25 % in der Arbeit mit Migrant*innen am niedrigsten. Fehlende gesellschaftliche Anerkennung („nicht anerkannt“, siehe Abb. 2) nehmen in der aktuellen Situation der Corona-Pandemie die nachfolgenden Handlungsfeldern wahr:

- Soziale Arbeit mit obdachlosen und/oder suchtkranken Menschen (77 %, *n* = 65),
- Hilfen zur Erziehung (77 %, *n* = 224)
- Soziale Arbeit mit Migrant*innen (74 %, *n* = 27),
- Kinder- und Jugendbildung (67 %, *n* = 534),
- Soziale Arbeit in Behörden (64 %, *n* = 124),
- Soziale Arbeit mit Arbeitslosen und/oder Sozialhilfeberechtigten (64 %, *n* = 22),
- mit Menschen mit Behinderung (62 %, *n* = 86) sowie
- im Gesundheitswesen und der Arbeit mit alten Menschen (61 %, *n* = 69).

Dabei steht die offizielle Zuschreibung der Systemrelevanz, also die Zuschreibung der Unverzichtbarkeit eines Handlungsfeldes für das Gemeinwesen, im Widerspruch zur Einschätzung der Befragten hinsichtlich der aktuell wahrgenommenen gesellschaftlichen Anerkennung des eigenen Arbeitsbereichs. Die Hintergrundannahme war hier, dass sich mit dem Status „systemrelevant“ möglicherweise auch die Tendenz zu einer entsprechend positive(ren) Wahrnehmung der gesellschaftlichen Anerkennung verzeichnen lässt^12^. Schließlich war bereits vor der Corona-Pandemie die unzureichende gesellschaftliche Anerkennung (sozial-)pädagogischer Berufsgruppen ein wichtiger Diskussionspunkt in der Professionsforschung (vgl. Schütz 2018; Schoneville und Thole 2009). Allerdings zeigt sich auch in der aktuellen Pandemie-Situation ein eher bekanntes Bild. Dies wird besonders am Beispiel der Hilfen zur Erziehung, der Kinder- und Jugendbildung, der Arbeit mit Migrant*innen sowie der Arbeit mit obdachlosen und/oder suchtkranken Menschen deutlich^13^. Diese Handlungsfelder werden zwar als systemrelevant eingestuft, die gesellschaftliche Anerkennung wird hier im Vergleich zwischen den Handlungsfeldern allerdings als gering wahrgenommen. Im Gegensatz zu den benannten Handlungsfeldern wurden die Beschäftigten etwa aus dem Bereich der Qualifizierung für die Soziale Arbeit nach eigenen Angaben offiziell nur zu einem kleinen Teil als systemrelevant eingestuft, dennoch fühlen sich die Befragten aus diesem Handlungsfeld sozial anerkannt. Ähnliches gilt für die Handlungsfelder Beratungsangebote sowie Soziale Arbeit im Gemeinwesen. Hier scheinen Tendenzen aus bereits vorhandenen Studien zur Anerkennung durch: Dabei sind Beschäftigte mit der eigenen gesellschaftlichen Anerkennung in Einrichtungen, die als „hochwertig“ erlebt werden (z. B. Hochschulen) zufriedener als andere (Schütz 2018, S. 188 f.).

Als weitere organisationale Rahmenbedingung in der aktuellen Arbeitssituation wurde auch das Vorhandensein notwendiger Schutzausrüstung erfragt. Die Beschäftigten in der Kinder- und Jugendbildung (37 %), im Bereich Hilfen zur Erziehung (14 %), in Behörden (9 %), Beratungsangeboten (5 %) und in der Arbeit mit Migrant*innen (2 %) gehören zu denjenigen Handlungsfeldern, denen es in der aktuellen Krise an Schutzausrüstung mangelt (*n* = 1126)^14^. Dabei zeigt sich ein signifikanter, wenn auch schwacher Zusammenhang zwischen der wahrgenommenen Anerkennung in der Gesellschaft und der zur Verfügung stehenden Ausrüstung zur Umsetzung von Hygienemaßnahmen^15^. Die Befragten, die nicht über genügend Schutzausrüstung verfügen, haben öfter angegeben sich nicht anerkannt zu fühlen (67 %), als diejenigen Befragten, die zum Zeitpunkt der Befragung genügend Schutzausrüstung zur Verfügung hatten (33 %). Darüber hinaus kann auch der angeforderte Sicherheitsabstand von 1,5 Metern insgesamt in nur der Hälfte der Einrichtungen (*n* = 1777) einhalten werden, wobei knapp 61 % der Befragten, deren Einrichtung für Mitarbeitende und Adressat*innen geöffnet ist (*n* = 1042) angeben, dass ein Sicherheitsabstand von dieser Größe nicht möglich sei. Dies scheint insbesondere die Hilfen zur Erziehung, den Bereich der Kinder- und Jugendbildung und die Arbeit mit Menschen mit Behinderung zu betreffen.

Die Maßnahmen zur Begegnung der Pandemie bringen nicht nur Widersprüche in der offiziellen und sozialen Anerkennung von Handlungsfeldern der Sozialen Arbeit ans Tageslicht, sie führen auch zu Veränderungen in der Zusammenarbeit mit Kolleg*innen und Adressat*innen. Zunächst zum Austausch mit den Kolleg*innen: Hier stellen 49 % der Teilnehmenden (*n* = 1810) im Zusammenhang mit der Pandemie eine Verringerung des fachlichen Austauschs fest (dies zeigt sich für nahezu alle Handlungsfelder), für 27 % hat er sich intensiviert und für 24 % ist er gleich geblieben. Damit sind insgesamt 76 % der Befragten durch die Corona-Pandemie und die damit verbundenen Einschränkungen in ihrem Austausch mit Kolleg*innen von Veränderungen betroffen^16^.

**Figure 3:**
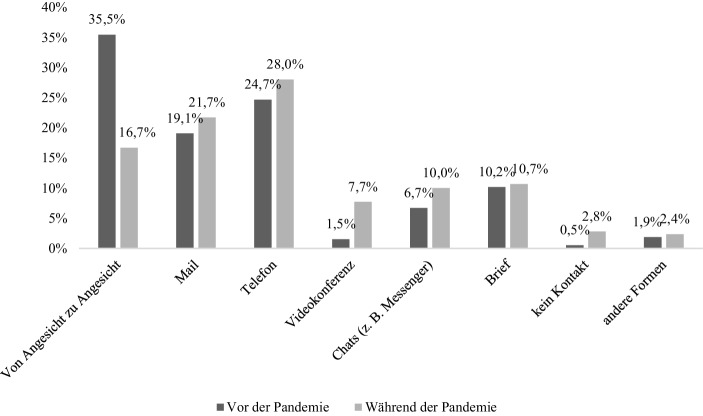

Veränderungen betreffen aber auch die Zusammenarbeit mit den Adressat*innen: Zunächst haben drei Viertel der Teilnehmenden der Befragung (*n* = 1849) angegeben, in den letzten sieben Tagen (zum Zeitpunkt der Befragung) Kontakt zu den Adressat*innen gehabt zu haben; ein Viertel hat dies verneint. Dabei fällt auf, dass es vor allem die Beschäftigten der Sozialen Arbeit in den Handlungsfeldern Hilfen zur Erziehung, im Gesundheitswesen und mit alten Menschen, mit Menschen mit Behinderung, mit Migrant*innen und mit obdachlosen und/oder suchtkranken Menschen sowie Beratungsangeboten waren, die Kontakt mit den Adressat*innen hatten^17^. Abb. 3 macht dabei über alle Handlungsfelder hinweg den subjektiv wahrgenommenen Unterschied in der Art der Kontaktaufnahme vor und während der Pandemie deutlich. Der Rückgang der Kontaktaufnahme von Angesicht zu Angesicht ist dabei aufgrund der Kontaktbeschränkungen nicht verwunderlich, diese waren allerdings für zahlreiche Handlungsfelder der Sozialen Arbeit keineswegs rechtlich angeordnet. Gerade aus dem Umstand der Kontaktnotwendigkeit ergibt sich u. a. die Systemrelevanz. Gleichwohl zeigt sich über die Handlungsfelder hinweg, dass insbesondere die Kontaktaufnahme per Video, Chat oder Telefon zugenommen hat. Von den Befragten sehen 34 % keine Schwierigkeiten darin, dass sich die Kontaktaufnahme zu den Adressat*innen verändert hat, zwei Drittel bewerten die Änderungen aber durchaus als problematisch (*n* = 1663). Dies sind insbesondere die Beschäftigten der Sozialen Arbeit in den Handlungsfeldern Soziale Arbeit in Behörden, Arbeit mit arbeitslosen und/oder Sozialhilfeberechtigten, mit Menschen mit Behinderung, mit obdachlosen und/oder suchtkranken Menschen sowie Beratungsangeboten^18^. Worin die Problematik liegt, wird von den Befragten (*n* = 964) in der daran anschließenden offen Frageoption konkretisiert, wobei unterschiedliche Aspekte betont werden. Diese reichen von der fehlenden persönlichen Einwirkungsmöglichkeit im Rahmen des Kinderschutzes bis hin zur nahezu banalen Erkenntnis, dass das Begleiten von Menschen in Krisensituationen eben nicht in Distanz möglich sei (vgl. Nittel und Meyer 2018). Eine befragte Person fasst dies so zusammen: „Am Telefon kann man nicht die gleiche Vertrauensbasis schaffen wie persönlich. Mit Kindern zu telefonieren ist schwierig. Hausbesuche, um sich ein Bild zu machen funktioniert nur vor Ort“. Und eine weitere Person konkretisiert diesen Gedanken: „Telefonische Beratung mit den Jugendlichen gestaltet sich schwierig. Sie befinden sich im Haushalt und machen möglicherweise derzeitige Probleme im Haushalt nicht offen“. Aber auch der Wegfall strukturschaffender Maßnahmen wie ein gemeinsames Abendessen erschwere den Adressat*innen den ohnehin oft problembelasteten Alltag zusätzlich und eröffne den Fachkräften damit einen weiteren ‚Krisenherd‘. Außerdem seien beispielsweise in den stationären Hilfen zur Erziehung die Einhaltung der notwendigen Hygienemaßnahmen einerseits unmöglich. Andererseits nähmen die Konflikte zwischen den Jugendlichen in solchen Einrichtungen durch das Kontaktverbot weiter zu. Benannt werden daneben auch ganz allgemein die zu verhindernden sozialen Folgen der Isolation von Adressat*innen sowie die damit verbundene Abnahme der Sichtbarkeit der Adressat*innen in der Gesellschaft.

Einen weiteren Eindruck dieser problematischen Veränderung des professionellen Handelns liefern auch die offenen Antworten (*n* = 410) am Ende des Fragebogens: Für die Adressat*innen brechen aus Sicht der Befragten wichtige Struktur- und Stützelemente des alltäglichen Lebens im Zuge des „Lockdowns“ weg. Darüber hinaus werden die Adressat*innen Sozialer Arbeit aus ihrer Sicht im öffentlichen Diskurs zu den angemessenen Maßnahmen zur Eindämmung der Pandemie weitgehend marginalisiert, obwohl gerade sie besonders stark von den Folgen betroffen gewesen seien. Die Aufrechterhaltung des Kontakts zu den Adressat*innen sei durch die Veränderung der Medien wie Telefon- oder Onlineangebote kaum möglich gewesen. Gleichzeitig verweisen einige Teilnehmende darauf, dass sich vor diesem Hintergrund die Kontaktzahl mit den Adressat*innen zum Teil deutlich reduziert und die Folgen (z. B. bei psychisch kranken Menschen mit ambulanter Anbindung) unklar sind.

Bei genauerer Betrachtung fällt darüber hinaus auch hier die ungleiche Verteilung der wahrgenommenen Veränderungen zwischen den Handlungsfeldern ins Auge. So empfinden besonders die Befragten aus den Handlungsfeldern Soziale Arbeit in Behörden (z. B. Allgemeiner Sozialer Dienst (ASD)), der Kinder- und Jugendbildung, der Sozialen Arbeit im Gesundheitswesen, der Sozialen Arbeit mit Menschen mit Behinderung sowie im Kontext von Beratungsangeboten die Veränderungen in der Kommunikation als problematisch.

Zwei Aspekte heben die Befragten in den verschiedenen offenen Antworten (*n* = 410) zudem immer wieder hervor:

1. *Die Corona-Pandemie und die Folgen verschärfen Arbeitsbedingungen in der Sozialen Arbeit: *Die Befragten beklagen einen fehlenden Schutz der Mitarbeitenden sowie die professionelle Unmöglichkeit in der eigenen Arbeit (z. B. in Kindertagesstätten oder stationären Wohnformen) angeordnete Hygienestandards zu befolgen. Dieser Umstand werde noch durch die erschwerte Kommunikation verstärkt, weil es aufgrund fehlender technischer Instrumente oder der Home Office-Regelungen schwer bis unmöglich sei, die eigenen Kolleg*innen oder Fachkräfte anderer Institutionen zu erreichen. Darüber hinaus habe in eigenen Handlungsfeldern die Kompensation durch den Wegfall der ehrenamtlichen Helfer*innen, die zumeist einer der so genannten Risikogruppen angehört haben, erhebliche personelle Ressourcen gebunden^19^. Auch berichten zahlreiche Befragte, dass sie zu fachfremder Arbeit verpflichtet worden seien, offenbar um Minusstunden zu vermeiden. Darüber hinaus verschärft sich die Arbeitssituation, neben der Arbeitsverdichtung in der Institution, durch die Notwendigkeit zur Betreuung der eigenen Kinder.
2. *Professionelle Handlungsweisen verändern sich in der Corona-Pandemie: *Befragte aus dem Bereich der Sozialen Arbeit in Behörden sowie von den Einrichtungen der Hilfen zur Erziehung berichten von der Verletzung professioneller Standards, um überhaupt arbeitsfähig zu bleiben oder unter den spezifischen Bedingungen überhaupt wieder Arbeitsfähigkeit zu erlangen. Aus diesem Grund beschreiben die Beschäftigten in den offenen Antworten die Wahrnehmung einer verstärkten Digitalisierung. Diese wird nun mit hoher Geschwindigkeit umgesetzt, weil die Gesamtsituation es nicht anders zu lässt. Parallel wird diese Entwicklung von den befragten Beschäftigten kritisch gesehen, weil die Adressat*innen nicht mehr angemessen erreicht werden können und sich auf diese Weise professionelle Handlungsweisen verändern. Für einige Befragte birgt die Corona-Pandemie in erster Linie die Option zu einem Digitalisierungsschub in der Sozialen Arbeit. Dies allerdings oft um den Preis der Verletzung von Datenschutzstandards sowie der „kopflosen“ Buchung von kommerziellen Leistungen Dritter ohne Wissen über die Folgen. Gerade dieser Umstand lenkt den Blick auf eine große Sorge der Befragten: Dass nach der Corona-Pandemie eine langfristige Änderung der Struktur Sozialer Arbeit stattgefunden hat und sich unkritisch neue Standards etabliert haben, die bisher als dysfunktional und ablehnungsbedürftig galten.

**Figure 4:**
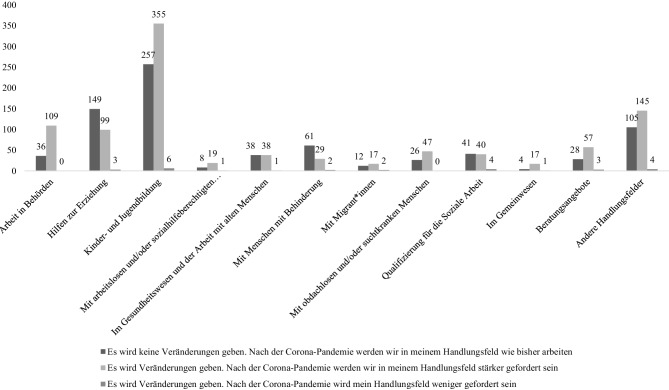

Abschließend noch ein kurzer Blick in die Zukunft: Hier sind 55 % der Meinung, dass das eigene Handlungsfeld nach der Pandemie stärker gefordert sein wird, 43 % glauben, dass das eigene Handlungsfeld genauso gefordert sein wird wie bisher und nur knapp 2 % sehen ein Zurückgehen der Anforderungen an das eigene Handlungsfeld. In Abb. 4 ist die Einschätzung der Befragten entsprechend der unterschiedlichen Handlungsfelder abgebildet. Dabei changieren die Wahrnehmungen sowohl zwischen den Handlungsfeldern als auch mit Blick auf das Antwortverhalten in der jeweiligen Gruppe. Dies ließe sich einerseits mit einer extremen Heterogenität innerhalb der Sozialen Arbeit sowie in den jeweiligen Handlungsfeldern deuten, ebenso aber mit Blick auf die unklare Lage im Zusammenhang mit der Corona-Pandemie.

In den offenen Antworten (*n* = 410) ist das Meinungsbild dagegen eindeutiger: Hier dominiert die Perspektive, dass die Folgen der Pandemie die Soziale Arbeit erst noch treffen werden. Dies gilt einerseits für die Entwicklungen auf Seiten der Adressat*innen, die Befragten rechnen beispielsweise mit einem Anstieg der Zahl von wohnungslosen Menschen ebenso wie mit einem Wachstum psychischer Erkrankungen. Andererseits sehen sich auch die Beschäftigten mit einer ungewissen Zukunft konfrontiert. Einige der befragten Beschäftigten freier Träger*innen sorgen sich besonders um ihre Zukunft (vgl. Bundesministerium für Arbeit und Soziales (BMAS) 2020).

## Zusammenfassung und Ausblick

Die gezeigten Trends der Befragungsergebnisse liefern einen ersten Eindruck davon, welche Auswirkungen die Corona-Pandemie aus Sicht der Beschäftigten für die Soziale Arbeit haben kann. Natürlich müssen weitere Analysen folgen. Unabhängig davon wird aber deutlich, dass die Corona-Pandemie auch aus Sicht der Beschäftigten beträchtlichen Einfluss auf die professionellen Arbeitsbeziehungen nimmt. Dies zeigt sich mit Blick auf die Sorge um die sich verschärfenden Lebensbedingungen der Adressat*innen Sozialer Arbeit, um die sich erschwerenden eigenen Arbeitsbedingungen sowie die Veränderung professionelle Handlungsweisen in Folge der Corona-Pandemie. Insgesamt fällt auf, dass sich – trotz partiell unterschiedlicher Schwierigkeiten oder Besonderheiten in einzelnen Bereichen – v. a. in den offenen Antworten doch recht homogene Einschätzungen und Wahrnehmungen zeigen. Hier kündigen sich für die befragten Personen in der aktuellen Lage Deprofessionalisierungstendenzen in der Sozialen Arbeit an (vgl. Meyer 2019). Immerhin vollzieht sich der wahrgenommene Wandel von Standards nicht auf Basis von Wissen aus der Sozialen Arbeit. Vielmehr werden Argumente aus anderen Sinnbereichen (Ökonomie: Arbeitszeit, Medizin: Schließung von Einrichtungen der Wohnungslosen zum Schutz der Mitarbeitenden) entlehnt und notwendigerweise auf die Soziale Arbeit angewendet. Eine allgemeine Aufwertung des Ansehens wird in der Folge gerade nicht wahrgenommen und das, obwohl einige Beschäftigte aktuell unter herausfordernden Bedingungen arbeiten: Immerhin sind, so wird notiert, einerseits Hygieneregeln durch den personenbezogenen Charakter der Arbeit kaum umsetzbar und andererseits fehlen noch mögliche Schutzinstrumente. Vor diesem Hintergrund wirkt die Unzufriedenheit mit der fehlenden gesellschaftlichen Anerkennung nicht allein als inhaltsleere Klage.

Zum Ausblick noch ein tiefergehender Blick in die Daten, der über erste Trends hinausgeht und aufzeigt, welche Veränderungen in Zukunft noch anstehen könnten. In den offenen Antworten zeigen sich aus professionstheoretischer Perspektive Veränderungen im antizipierten Mandat. So gibt es in den offenen Antworten Berichte über die Versetzung zur Stadtpolizei, um hier auf Corona-Streife zu gehen. Daneben berichten Befragte davon, dass sie relevante Angebote für die Adressat*innen nicht mehr durchführen können, weil die Institutionen die Durchsetzung der Hygieneregeln als bedeutsamer begreifen. Aus professioneller Perspektive schätzen die Befragten hier den Sachverhalt zumeist anders ein. Auch in der Corona-Pandemie müssen für bestimmte Gruppen weiter Angebote vorhanden sein. In solchen berufsethischen Argumentationen im Rahmen der offenen Antworten ist den Teilnehmenden besonders wichtig, dass sie zu keinem Zeitpunkt die eigene gesundheitliche Sicherheit vor das Wohl der Adressat*innen stellen würden. Diese starke berufsbiografische Basisposition findet entsprechend ihren Ausdruck im Ärger über das gesellschaftliche, mediale und politische Vorgehen: So kritisieren die Befragten in den offenen Antworten das Reden ‚über‘ die Soziale Arbeit und ihre Adressat*innen als Ausdruck der Sprachlosigkeit von Disziplin und Profession sowie der geringen Wertschätzung der Gesellschaft gegenüber der Sozialen Arbeit aus.

Die sozialen Folgen der Corona-Pandemie werden, nicht nur angesichts der vorliegenden Befragungstrends, die Soziale Arbeit vermutlich erst in den kommenden Monaten treffen. Vor diesem Hintergrund werden die durch die Fachkräfte der Sozialen Arbeit in der Befragung wahrgenommenen Veränderungen in den Arbeitsbündnissen der Sozialen Arbeit weiter zu beobachten sein. Gerade in den offenen Antworten hat sich bereits eine Verschiebung des Mandats ebenso wie die große Sorge vor den wirtschaftlichen Folgen gerade freier Träger angedeutet^20^.

## Footnotes

1 Harold L. Wilensky (1972) beschreibt drei zentrale Phasen in der Herausbildung einer Profession: 1) Die Zusammenfassung einer Berufsfunktion zu einem Hauptberuf. 2) Die Schaffung einer wissenschaftlich fundierten Ausbildung. 3) Die Gründung von Berufsverbänden, die diesen letzten Schritt der Professionalisierung markiert: „Bedingung für die Möglichkeit einer wirksamen öffentlichen Interessenartikulation ist […] die Existenz eines Berufsverbandes“ (Nittel 2000, S. 64).

2 Hier wird die Zersplitterung der zahlreichen Berufs‑, Fach- oder Interessenverbände deutlich. So gelangen nur die Positionen einzelner gut vernetzter Verbände an die (mediale) Öffentlichkeit. Eine Position ‚der‘ Sozialen Arbeit wird damit nicht deutlich und in der Folge auch nicht von Gesellschaft oder Politik wahrgenommen.

3 Diese Beziehung konstituiert sich für Ulrich Oevermann (1996) als Balance von diffuser sowie spezifischer Sozialbeziehung und ist als Erklärungsmodell professionalisierten Handelns in der Sozialen Arbeit weitgehend etabliert (vgl. Müller-Hermann 2020).

4 Für die Erstellung des Fragebogens wurde Sosci Survey verwendet. Der Fragebogen wurde aus Zeitgründen einem standardisierten und keinem qualitativen Pretest unterzogen (*n* = 6), bestand aus 48 Fragen (inkl. Filterfragen) und dauerte im Schnitt 12 min.

5 Dazu gehörten: Vereinte Dienstleistungsgewerkschaft (ver.di), DBSH, Verteiler Junge Wissenschaft der Sozialen Arbeit der DGfE (JuWiSozA) sowie Professuren im Bereich Soziale Arbeit an unterschiedlichen Hochschulen.

6 Insgesamt haben 2344 Personen den Fragebogen begonnen, knapp 20 % von diesen haben den Fragebogen (insbesondere im ersten Drittel) abgebrochen.

7 Mit 14,6 % sind auch andere Handlungsfelder stark vertreten. Darunter gefasst sind Handlungsfelder unter 1 % (wie z. B. Frauenhäuser) sowie die Option „keines der genannten Handlungsfelder“.

8 Die Frage lautete: „Systemrelevante Berufe“ meint jene Berufe, die als grundsätzlich unverzichtbar für das Gemeinwesen gelten. Die Liste der systemrelevanten Berufe und Berufsgruppen wird von den Bundesländern geführt und kann variieren. Wird Ihre Tätigkeit als „systemrelevant“ eingestuft, d. h. Sie bekommen beispielsweise eine Notbetreuung minderjähriger Kinder trotz der verfügten Schließung von Schulen und Kindertagesstätten?.

9 Das Handlungsfeld und die Einstufung als systemrelevant stehen in einem statistisch signifikanten Zusammenhang (*X*^*2*^*(11)* = 177,63, *p* = 0,000, *n* = 1577), mit einem mittleren Effekt (*CC* = 0,318, *p* = 0,000; Cramers *V* = 0,336, *p* = 0,000).

10 Die Frage lautete: Fühlen Sie sich in Ihrer Tätigkeit durch die Menschen in der Gesellschaft aktuell anerkannt?.

11 Das Handlungsfeld und die Bewertung der gesellschaftlichen Anerkennung stehen in einem statistisch signifikanten Zusammenhang (*X*^*2*^*(11)* = 113,27, *p* = 0,000, *n* = 1539), mit einem geringen Effekt (*CC* = 0,262, *p* = 0,000; Cramers *V* = 0,271, *p* = 0,000).

12 Annahme ist dabei nicht, dass gesellschaftliche Anerkennung ausschließlich vom Status der Systemrelevanz abhängt, noch Systemrelevanz zu mehr Anerkennung führt. Der Status der Systemrelevanz wird vielmehr als möglicher Aspekt vielfältiger Arbeitsbedingungen betrachtet (z. B. Einkommen, Anstellungsverhältnis, organisationale Rahmenbedingungen), die Einfluss auf die subjektive Wahrnehmung gesellschaftlicher Anerkennung nehmen.

13 In den als systemrelevant eingestuften Handlungsfeldern zeigt sich zwischen den Handlungsfeldern und der Bewertung der gesellschaftlichen Anerkennung ein statistisch signifikanter Zusammenhang (*X*^*2*^*(11)* = 53,70, *p* = 0,000, *n* = 742), mit einem geringen Effekt (*CC* = 0,260, *p* = 0,000; Cramers *V* = 0,269, *p* = 0,000).

14 Das Handlungsfeld und das Vorhandensein von Schutzausrüstung stehen in einem statistisch signifikanten Zusammenhang (*X*^*2*^*(11)* = 34,67, *p* = 0,000, *n* = 1607), mit einem geringen Effekt (*CC* = 0,145, *p* = 0,000; Cramers *V* = 0,147, *p* = 0,000).

15 Anerkennung in der Gesellschaft und Vorhandensein von Schutzausrüstung stehen in einem signifikanten Zusammenhang (*X*^*2*^*(19,58)* = 34,67*, p* = 0,000, *n* = 1350), mit einem geringen Effekt (*φ* = 0,120, *p* = 0,000).

16 Das Handlungsfeld und der Austausch mit Kolleg*innen stehen in einem statistisch signifikanten Zusammenhang (*X*^*2*^*(22)* *=* *54,59, p* = 0,000, *n* = 1810), mit einem geringen Effekt (*CC* = 0,171, *p* = 0,000; Cramers *V* = 0,123, *p* = 0,000).

17 Das Handlungsfeld und der Kontakt mit den Adressat*innen stehen in einem statistisch signifikanten Zusammenhang (*X*^*2*^*(11)* *=* *116,05, p* = 0,000, *n* = 1849), mit einem geringen Effekt (*CC* = 0,243, *p* = 0,000; Cramers *V* = 0,251, *p* = 0,000).

18 Das Handlungsfeld und die Bewertung der Kontaktaufnahme stehen in einem statistisch signifikanten Zusammenhang (*X*^*2*^*(11)* *=* *47,80, p* = 0,000, *n* = 1663), mit einem geringen Effekt (*CC* = 0,167, *p* = 0,000; Cramers *V* = 0,170, *p* = 0,000).

19 Aus professionstheoretischer Perspektive ist dieser Umstand von ungeheurem Interesse. Immerhin zeigt er, dass *ehrenamtliche* Personen nur unzureichend *beruflich* erbrachte Leistungen kompensieren können.

20 Das Sozialdienstleister-Einsatzgesetz ist aus professionstheoretischer Perspektive brandgefährlich: Statt die wichtige Funktion der Sozialen Arbeit und der in ihr Beschäftigten für die soziale Stabilität von Menschen sowie damit der Gesellschaft anzuerkennen, können Beschäftigte von Trägern unter diesem „Rettungsschirm“ zum Ernteeinsatz oder dem Füllen von Supermarktregalen herangezogen werden.
